# 5-Hydroxytryptamine Receptors and Tardive Dyskinesia in Schizophrenia

**DOI:** 10.3389/fnmol.2020.00063

**Published:** 2020-04-24

**Authors:** Ivan V. Pozhidaev, Diana Z. Paderina, Olga Yu. Fedorenko, Elena G. Kornetova, Arkadiy V. Semke, Anton J. M. Loonen, Nikolay A. Bokhan, Bob Wilffert, Svetlana A. Ivanova

**Affiliations:** ^1^Mental Health Research Institute, Tomsk National Research Medical Center of the Russian Academy of Sciences, Tomsk, Russia; ^2^Department of Cytology and Genetics, National Research Tomsk State University, Tomsk, Russia; ^3^Division for Control and Diagnostics, School of Non-Destructive Testing and Security, National Research Tomsk Polytechnic University, Tomsk, Russia; ^4^Hospital, Siberian State Medical University, Tomsk, Russia; ^5^PharmacoTherapy, -Epidemiology and -Economics, Groningen Research Institute of Pharmacy, University of Groningen, Groningen, Netherlands; ^6^Policy Office for Quality and Innovation of Care (BZI), GGZ Westelijk Noord-Brabant, Halsteren, Netherlands; ^7^Department of Psychiatry, Addictology and Psychotherapy, Siberian State Medical University, Tomsk, Russia; ^8^Department of Psychotherapy and Psychological Counseling, National Research Tomsk State University, Tomsk, Russia; ^9^Clinical Pharmacy and Pharmacology, University Medical Center Groningen, University of Groningen, Groningen, Netherlands

**Keywords:** schizophrenia, tardive dyskinesia, 5-HT receptors, pharmacogenetics, gene polymorphisms

## Abstract

**Background:**

Tardive dyskinesia (TD) is a common side effect of antipsychotic treatment. This movement disorder consists of orofacial and limb-truncal components. The present study is aimed at investigating the role of serotonin receptors (HTR) in modulating tardive dyskinesia by genotyping patients with schizophrenia.

**Methods:**

A set of 29 SNPs of genes of serotonin receptors *HTR1A, HTR1B, HTR2A, HTR2C, HTR3A, HTR3B*, and *HTR6* was studied in a population of 449 Caucasians (226 females and 223 males) with verified clinical diagnosis of schizophrenia (according to ICD-10: F20). Five SNPs were excluded because of low minor allele frequency or for not passing the Hardy-Weinberg equilibrium test. Affinity of antipsychotics to 5-HT2 receptors was defined according to previous publications. Genotyping was carried out with SEQUENOM Mass Array Analyzer 4.

**Results:**

Statistically significant associations of rs1928040 of *HTR2A* gene in groups of patients with orofacial type of TD and total diagnosis of TD was found for alleles, and a statistical trend for genotypes. Moreover, statistically significant associations were discovered in the female group for rs1801412 of *HTR2C* for alleles and genotypes. Excluding patients who used HTR2A, respectively, HTR2C antagonists changed little to the associations of *HTR2A* polymorphisms, but caused a major change of the magnitude of the association of *HTR2C* variants. Due to the low patient numbers, these sub-analyses did not have significant results.

**Conclusion:**

We found significant associations in rs1928040 of *HTR2A* and for rs1801412 of X-bound *HTR2C* in female patients. The associations were particularly related to the orofacial type of TD. Excluding patients using relevant antagonists particularly affected rs1801412, but not rs1928040-related associations. This suggest that rs1801412 is directly or indirectly linked to the functioning of HTR2C. Further study of variants of the *HTR2C* gene in a larger group of male patients who were not using *HTR2C* antagonists is necessary in order to verify a possible functional role of this receptor.

## Introduction

The abnormal involuntary movement disorder tardive dyskinesia (TD) is a common side effect of both first- and second-generation antipsychotic drugs (Tenback and van Harten, 2011; Carbon et al., 2017; Widschwendter and Hofer, 2019). The clinical picture and course of TD is not unambiguously defined (Loonen et al., 2019) and several other extrapyramidal movement reactions occur simultaneously in patients treated with antipsychotic drugs (Loonen et al., 2000, Loonen et al., 2001). These other drug-induced movement disorders may also have a tardive course and often accompany TD in fluctuating intensity which could make it hard to correctly diagnose a case with dyskinesia in epidemiological studies (Waln and Jankovic, 2013; D’Abreu et al., 2018). Dyskinesia is known to occur spontaneously, particularly in elderly persons (Woerner et al., 1991; Clark and Ram, 2007), and also in drug-naïve persons with schizophrenia (Kane and Smith, 1982; Pappa and Dazzan, 2009) as well as in their direct relatives (McCreadie et al., 2003; Koning et al., 2010). This spontaneously occurring dyskinesia may obscure the prevalence of truly drug-induced movement disorders. Trying to identify biomarkers which could predict vulnerability to develop TD in individual patients using epidemiological data as starting point is probably problematic in the yielding of fruitful results. Pharmacogenetic information can better be applied to help clarify the possible pharmacological mechanisms required in the explanation of the pathogenesis of the movement disorder, and/or the modification of the intensity of its symptoms (Loonen et al., 2019).

TD is characterized by involuntary repetitive movements which are usually abrupt and irregular in nature (Loonen and van Praag, 2007). The movements may affect tongue, lips and jaw as well as the forehead, eyelids (blinking), lower face and throat (orofacial dyskinesia); the neck, trunk (rocking movements) and upper and lower limbs may also show rapid repetitive contractions (peripheral or limb-truncal dyskinesia). When the diaphragm and intercostal muscles are affected, dyskinetic movements result in making grumbling, snoring, groaning, and/or sniffing noises (respiratory dyskinesia) (Van Harten and Tenback, 2011). This last type is usually not considered in epidemiological or treatment studies. In the earliest reports about TD, this movement disorder was characterized by involuntary orofacial movements (Loonen et al., 2019). This should be considered to be “classic” TD (Waln and Jankovic, 2013). We have observed that different gene variants are associated with orofacial versus limb-truncal dyskinesia (Al Hadithy et al., 2009, 2010). We have also noticed that in levodopa-induced dyskinesia, specific genetic associations exist with limb-truncal dyskinesia (LID), but not with orofacial dyskinesia (Ivanova et al., 2012). This may correspond with the observation that in Huntington’s disease (HD) and LID, large muscle groups are often affected, while in TD more subtle movements are present, most often in the orofacial area. We could imagine that in TD the dysregulation is primarily localized within another histological striatal compartment than in HD and LID (Loonen et al., 2019).

TD most likely results from dysregulation within so-called dorsal extrapyramidal cortico-striatal-thalamic-cortical (CSTC) circuits (Loonen and Ivanova, 2013; Loonen et al., 2019). CSTC circuits comprising the putamen as striatal entry station to the basal ganglia regulate the intensity (amplitude and velocity) of voluntary muscle contractions. The activity of these CSTC circuits is in turn primarily regulated by ascending dopaminergic nigrostriatal neurons. However, ascending serotonergic input modulates the activity of CSTC circuits too (Loonen and Ivanova, 2016; Loonen et al., 2019). 5-Hydroxytryptamine (5-HT, serotonin)-containing fibers originating within brainstem upper raphe nuclei have a widespread distribution within the midbrain and forebrain (Loonen and Ivanova, 2016). They are heavily connected with, for example, the substantia nigra pas compacta (SNc), the dorsal striatum, and the frontal cerebral cortex, next to many other structures. By affecting several components of the CSTC circuits, they modulate their activity (Loonen and Ivanova, 2016). Moreover, they affect the activity of CSTC circuits indirectly via striatal interneurons and by modulating the stress response (Loonen et al., 2019). Seven types of 5-HT receptors (HTRs) can be distinguished, most of them having several subtypes. All but one (HTR3) are g-protein coupled receptors (GPCRs) (Hannon and Hoyer, 2008; Loonen and Ivanova, 2016). For their role in TD and LID, HTR1A, HTR2A and HTR2C have been most extensively studied (Meltzer, 2012; Huot et al., 2013). HTR1A are inhibitory (receptors coupled to Gi/o) and HTR2 are excitatory (receptors coupled to Gq/11) receptors. A special characteristic of HTR2C and, to lesser extent, HTR2A, is constitutive activity (Hannon and Hoyer, 2008). This offers the possibility of certain atypical antipsychotic drugs having inverse agonistic activity (Aloyo et al., 2009), which means that they affect HTR2 in a direction opposite to 5-HT itself. This complicates pharmacogenomic studies because it is not known whether a genetic variant of the *HTR2* gene affects the activity, the expression, the inducibility or the constitutive activity of the corresponding receptors. Therefore, an urgent need exists to develop an *in vivo* or *ex vivo* test suitable to assess the activity of receptor complexes corresponding to specific *HTR2* variants (Ivanova et al., 2018). Moreover, the use of drugs with inverse agonistic activity, like atypical antipsychotics, could obscure the possible consequences of HTR2 inactivity (Loonen et al., 2019).

The present study is aimed to investigate the possible role of serotonin receptors in tardive dyskinesia and its subtypes in patients with schizophrenia by studying associations with specific variants of *HTR* genes.

## Materials and Methods

### Patients

The present study was carried out in accordance with The Code of Ethics of the World Medical Association (Declaration of Helsinki 1975, revised in Fortaleza, Brazil, 2013) for experiments involving humans and after the study having been approved (protocol N63/7.2014) by the Local Bioethics Committee of the Mental Health Research Institute. Participants providing written informed consent were recruited from three psychiatric hospitals in Tomsk, Kemerovo and Chita oblasts in Siberia.

The study population was previously described (Boiko et al., 2019; Levchenko et al., 2019). Patients were recruited who were, or had been, using antipsychotic medication for more than 3 months and were on a stable dosage for at least 3 months prior to entry. The inclusion criteria were a clinical diagnosis of schizophrenia according to ICD-10 (F20) and an age of 18–75 years. Exclusion criteria were non-Caucasian physical appearance (e.g., Mongoloid, Buryats or Khakassians); any relevant (e.g., unstable or acute) physical disorders, relevant pharmacological withdrawal symptoms or organic brain disorders (e.g., epilepsy, Parkinson’s disease). The severity of dystonia was assessed with the Abnormal Involuntary Movement Scale (AIMS) (Loonen et al., 2000; Loonen et al., 2001; Loonen and van Praag, 2007). The AIMS scores were converted into binary form (presence or absence of tardive dyskinesia) according by Schooler and Kane’s criteria (Schooler and Kane, 1982). Schooler–Kane criteria require: (i) at least 3 months of cumulative exposure to neuroleptics; (ii) the absence of other conditions that might cause involuntary movements and (iii) at least moderate dyskinetic movements in one body area (≥3 on AIMS) or mild dyskinetic movements in two body areas (≥2 on AIMS). To compare antipsychotic medications, all drug doses taken at the time of investigation were converted into chlorpromazine equivalents (CPZeq) (Andreasen et al., 2010). In order to exclude a possible influence of drug-induced receptor inactivation on TD scores, patients using 5-HT2A and/or 5-HT2C receptor blocking antipsychotics (according to Loonen and Ivanova, 2016) were excluded in sub-analysis.

### DNA Analysis

Blood samples were obtained from each participant by antecubital venepuncture. Blood with EDTA was stored in several aliquots at −20°C until DNA isolation. DNA from leukocytes of whole peripheral blood was isolated according with standard phenol-chloroform protocol. Genotyping was performed without any knowledge of the patient’s clinical status. Genotyping was carried out for 29 polymorphic variants of *HTR1A* (rs6295, rs1364043, rs10042486, rs1800042, rs749099), *HTR1B* (rs6298, rs6296, rs130058), *HTR2A* (rs6311, rs6313, rs6314, rs7997012, rs1928040, rs9316233, rs2224721), *HTR2C* (rs6318, rs5946189, rs569959, rs17326429, rs4911871, rs3813929, rs1801412, rs12858300), *HTR3A* (rs1062613, rs33940208, rs1176713), *HTR3B* (rs1176744) and *HTR6* (rs1805054) on the MassARRAY Analyzer 4 (Agena Bioscience). Rs1800042, rs6314, rs12858300, rs33940208, and rs1176744 had a minor allele frequency less than 5% or did not pass Hardy–Weinberg equilibrium test (*p* < 0.05) and were excluded from analysis. We used the set SEQUENOM Consumables iPLEX Gold 384. DNA sample preparation for SEQUENOM MassARRAY Analyzer 4 includes several steps, including standard PCR reaction, a shrimp alkaline phosphatase reaction to neutralize the unincorporated dNTPs in the amplification products, the PCR iPLEX Gold extension reaction, and then placing the samples on a special chip (SpectroCHIP array) using Nanodispenser RS1000 prior to loading them into the analyser. DNA concentrations were measured with a Thermo Scientific NanoDrop 8000 UV-Vis Spectrophotometer. Possibly relevant SNPs were selected according to the literature data on associations with schizophrenia and other mental disorders and program LD TAG SNP Selection (TagSNP). More detailed information about selected SNPs are presented in Supplementary Table S1.

### Statistical Analysis

Statistical analysis was performed in the R statistical environment using basic R functions, PredictABEL and the SNPassoc package (Gonzalez et al., 2014). We conducted the statistical analysis in a few steps. Firstly, we tested all polymorphic variants to deviation from Hardy-Weinberg equilibrium with chi-square test, except those variants which were located in X-chromosome (*HTR2C* polymorphic variants). Polymorphic variants of *HTR2C* were divided by sex and tested separately because of hemizygotic status of X-chromosomal markers for men. In addition, polymorphic variants with minor allele frequency less than 5% were excluded from further testing. Secondly, we conducted association analysis of genotypes and alleles with tardive dyskinesia and its orofacial (AIMS item 1–4) and limb-truncal (AIMS items 5–7) subtypes with chi-square test and Fischer’s exact test, where necessary. Odds ratio and 95% confidence intervals (significance level was 0.05) were also tested. The third step was to assess variables through binary logistic regression.

## Results

The total number of patients was 449 (226 females and 223 males). According to predefined criteria the total number of patients with tardive dyskinesia was 121, thus the number of patients without tardive dyskinesia was 328. The main demographic and clinical parameters are presented in Table 1. The mean age of patients with tardive dyskinesia (patients with TD) was significantly higher than the age of patients without tardive dyskinesia (patients without TD), and the duration of schizophrenia was significantly longer in patients with TD and higher doses of antipsychotics were used in patients with TD. One hundred and ninety four patients received typical antipsychotics (of which Haloperidol was the most often used – 115 patients, Chlorpromazine – 44 patients, as well as Chlorprothixene, Zuclopenthixol, Thioridazine, Periciazine). 172 patients received atypical antipsychotics (mainly Risperidone, Clozapine and Quetiapine and to a lesser extent Olanzapine, Sertindole, Paliperidone, Amisulpride); 83 patients received combined therapy.

**TABLE 1:** Main demographic and clinical parameters of studied groups.

Diagnostic parameter	Patients without TD	Patients with TD	*P*-value
Total number of patients	328	121	–
Gender, n	Male –152 (46.3%) Female – 176 (53.7%)	Male – 71 (58.7%) Female – 50 (41.3%)	0.020
Median age [Q1; Q3], years	37[31; 48]	48[37.5; 58]	<0.001
Median age of onset [Q1; Q3], years	24[20; 30]	25[20; 32]	0.974
Median duration of illness [Q1; Q3], years	11[5; 18]	20[12;29.5]	<0.001
Median CPZeq [Q1; Q3], dose	396[200; 750]	500[286.2; 750]	0.021

*[Q1; Q3] – [first quartile; third quartile].*

The first step was to estimate a possible association between the autosomal genotypes or alleles concerning *HTR* genes and the presence or absence of total (Supplementary Table S2), orofacial (Supplementary Table S3) or limb-truncal (Supplementary Table S4) tardive dyskinesia. Almost all *HTR* variants showed no association with TD or one of its subtypes. An exception was formed by rs1928040 of the *HTR2A* gene. We found a significant association of the T allele with a total diagnosis of tardive dyskinesia (*p* = 0.02) and with orofacial TD (*p* = 0.02). The T allele was also associated with limb-truncal TD, but this association did not reach statistical significance (*p* = 0.07). The same was true for the association of the genotypes with these first two forms of TD (*p* = 0.061; *p* = 0.058, respectively) (Table 2). Excluding patients who were treated with 5-HT2A antagonists according to Loonen and Ivanova (2016) or periciazine (*N* = 185) hardly changed the calculated ORs of possible associations, although significance was lost due to the lower patient numbers and the resulting loss of statistical power (data not shown).

**TABLE 2:** Results of association analysis for genotypes and alleles of rs1928040 between groups of patients with different types of tardive dyskinesia and without tardive dyskinesia.

SNP/TD type	Genotypes/alleles	Patients with TD, %	Patients without TD, %	OR		χ^2^	p
				Value	95% CI		
rs1928040/orofacial TD	TT	29 (26.9%)	50 (16.8%)	1.81	1.07–3.06	5.687	0.058
	TC	47 (43.5%)	134 (45.1%)	0.94	0.60–1.46		
	CC	32 (29.6%)	113 (38.0%)	0.69	0.43–1.10		
	T	0.486	0.394	1.46	1.06–1.99	5.53	**0.02**
	C	0.514	0.606	0.69	0.50–0.94		
rs1928040/limbtruncal TD	TT	18 (28.1%)	61 (17.9%)	1.80	0.97–3.31	3.795	0.150
	TC	27 (42.2%)	154 (42.2%)	0.89	0.52–1.52		
	CC	19 (29.7%)	126 (37.0%)	0.72	0.40–1.29		
	T	0.492	0.405	1.43	0.98–2.08	3.39	0.07
	C	0.508	0.595	0.70	0.48–1.02		
rs1928040/total TD	TT	31 (25.8%)	48 (16.8%)	1.72	1.03–2.87	5.601	0.061
	TC	54 (45.0%)	127 (44.6%)	1.02	0.66–1.56		
	CC	35 (29.2%)	110 (38.6%)	0.66	0.41–1.04		
	T	0.483	0.391	1.46	1.07–1.97	5.89	**0.02**
	C	0.517	0.609	0.69	0.51–0.93		

*Significancy (p < 0.05) indicated in bold.*

The second step was to assess possible sex difference for X-bound *HTR2C* variants (Supplementary Tables S5–S7). To our surprise, we observed no association between any of the *HTR2C* polymorphisms and total, orofacial or limb-truncal TD in (hemizygous) males. In women, however, both genotypes and alleles of *HTR2C* polymorphism rs1801412 were significantly associated with total (*p* = 0.027, respectively, *p* = 0.03) and orofacial TD (*p* = 0.008; *p* = 0.009). Limb-truncal TD was not associated with this polymorphism for women (Table 3). Excluding patients who used 5-HT2C antagonists changed the measured ORs. In 141 female patients, the observed association decreased for total and orofacial TD, but in 102 male patients it increased for all three types of TD (Supplementary Tables S8, S9). Due to the low patient number, a statistical trend (*p* = 0.08) was reached only for orofacial TD in male patients.

**TABLE 3:** Results of association analysis for genotypes and alleles of rs1801412 between groups of patients with different types of tardive dyskinesia and without tardive dyskinesia after dividing by sex.

SNP/TD type	Genotypes/alleles	Patients with TD, %	Patients without TD, %	OR		χ^2^	*p*-value
				Value	95% CI		
rs1801412/female/orofacial TD	GG	0 (0.0%)	0 (0.0%)	4.32	0.08–220.72	7.138	**0.008**
	GT	7 (16.7%)	9 (4.9%)	3.87	1.35–11.08		
	TT	35 (83.3%)	174 (95.1%)	0.26	0.09–0.74		
	G	0.083	0.025	3.61	1.30–9.98	6.88	**0.009**
	T	0.917	0.975	0.28	0.10–0.77		
rs1801412/female/limb-truncal TD	GG	0 (0.0%)	0 (0.0%)	6.93	0.13–356.19	0.629	0.428
	GT	3 (10.7%)	13 (6.6%)	1.70	0.45–6.38		
	TT	25 (89.3%)	184 (93.4%)	0.59	0.16–2.21		
	G	0.054	0.033	1.66	0.46–6.01	0.61	0.44
	T	0.946	0.967	0.60	0.17–2.19		
rs1801412/female/total TD	GG	0 (0.0%)	0 (0.0%)	3.57	0.07–182.00	4.882	**0.027**
	GT	7 (14.3%)	9 (5.1%)	3.09	1.09–8.79		
	TT	42 (85.7%)	167 (94.9%)	0.32	0.11–0.92		
	G	0.071	0.026	2.93	1.06–8.08	4.70	**0.03**
	T	0.929	0.974	0.34	0.12–0.94		

*Significancy (p < 0.05) indicated in bold.*

The third step was to assess variables through binary logistic regression (Table 4). We used the status of types of tardive dyskinesia as the dependent variable, and “age,” “gender,” “duration of disease” and significant polymorphic variant rs1928040 as predictors. For orofacial TD Hosmer–Lemeshow test, the result is chi-square = 6.627, *p* = 0.5773 (*df* = 8); AUC [95% CI] for orofacial TD model is 0.752 [0.698–0.806]. For limb-truncal TD Hosmer-Lemeshow test, it is chi-square = 5.86, *p* = 0.6629 (*df* = 8); AUC [95% CI] for limb-truncal TD model is 0.700 [0.631–0.769]. For total diagnosis of TD Hosmer-Lemeshow test, the result is chi-square = 8.674, *p* = 0.3705 (*df* = 8); AUC [95% CI] for total diagnosis of TD model is 0.746 [0.693–0.800].

**TABLE 4:** Variables related with different types of tardive dyskinesia analyzed by binary logistic regression.

Type of tardive dyskinesia	Category	OR	95% CI lower bound	95% CI upper bound	*p*-value
Orofacial TD	rs1928040	0.6164	0.4366	0.8702	0.0060
	Gender	0.2670	0.1514	0.4708	<0.0001
	Age	1.0245	0.9966	1.0532	0.0856
	Duration of disease	1.0615	1.0296	1.0945	0.0001
Limb-truncal TD	rs1928040	0.6739	0.4585	0.9903	0.0445
	Gender	0.4407	0.2385	0.8143	0.0089
	Age	1.0278	0.9960	1.0606	0.0874
	Duration of disease	1.0345	1.0010	1.0693	0.0437
Total TD	rs1928040	0.6219	0.4453	0.8685	0.0053
	Gender	0.3038	0.1767	0.5224	<0.0001
	Age	1.0236	0.9967	1.0511	0.0856
	Duration of disease	1.0619	1.0308	1.0940	0.0001

The present study shows good values of AUC, which indicates approximately good-fitted regression models for different types of tardive dyskinesia in patients with schizophrenia. It revealed that, compared with group of patients without TD, patients with TD have significantly increased odds in variable “Duration of disease” and significantly decreased in variables “Gender” and “rs1928040” (Table 4).

## Discussion

The present paper describes the results of an association study of in the end 24 polymorphic variants of *HTR1A*, *HTR1B*, *HTR2A, HTR2C*, *HTR3A*, *HTR3B*, and *HTR6* genes with TD and two of its subtypes in 449 patients with schizophrenia. As *HTR2C* is X-bound the possible association was studied in male and female patients independently. We found a significant association of the T allele of rs1928040 of the *HTR2A* gene with total and orofacial TD. In addition, both genotypes and alleles of *HTR2C* polymorphism rs1801412 was significantly associated with total and orofacial TD in women, but not in men.

Rs1928040 is a C > T intron 2 variant of *HTR2A* gene which is localized on chromosome 13. This variant has been reported to be associated with the response to treatment with selective serotonin uptake inhibitors (SSRIs) in patients with major depressive disorders, but the results are inconsistent (Kishi et al., 2010; Lucae et al., 2010; McMahon et al., 2006). Rs1801412 is a T/G on position 114908141 of the *HTR2C* gene on the X-chromosome. To our knowledge, an association with central nervous system disorders has not been observed (Serretti et al., 2009; Bakker et al., 2012); this includes antipsychotic-induced movement disorders in a Dutch population of long-stay psychiatric patients (Bakker et al., 2012).

An essential component of the pathogenesis of tardive dyskinesia (Loonen and Ivanova, 2013) as well as levodopa-induced dyskinesia (Ivanova and Loonen, 2016) is excitotoxic damage to striatal medium spiny projection neurons (MSNs) of the indirect extrapyramidal pathway (Loonen et al., 2019). This slow damage could explain the late onset of the movement disorder and the resulting dominance of dopamine D1 carrying MSNs of the direct pathway, which is essential for mediating dyskinesia (Westin et al., 2007; Darmopil et al., 2009). We have hypothesized that second generation antipsychotics (SGAs) can protect indirect pathway MSNs against excitotoxicity by inverse agonism of HTR2A and particularly HTR2C which these neurons carry (Loonen and Ivanova, 2016; Loonen et al., 2019). Modulation of the activity of HTR2A or HTR2C resulting from genetic variability could then also result in differences in the incidence of tardive dyskinesia during exposure to antipsychotic drugs. In addition, fast spiking interneurons which inhibit the activity of striatal dopaminergic terminals also express HTR2A or HTR2C (Loonen and Ivanova, 2016; Loonen et al., 2019). Inverse agonism by SGAs would increase the release of dopamine, which could directly stimulate direct pathway MSNs causing acute dyskinesia. Hence, present usage of HTR2A or HTR2C antagonists could obscure a possible genetically induced change in prevalence.

In our statistical analysis, both rs1928040 and rs1801412 were linked more intensively to orofacial than to limb-truncal TD. This corresponds to our previous observations that the genetic background of these two types of TD may be different (Al Hadithy et al., 2009, 2010). It has been suggested that the orofacial variant corresponds to “classical” TD (Waln and Jankovic, 2013) and is possibly related to a disfunction within another striatal tissue compartment (i.e., striosomal) than HD and LID (i.e., matrix) (Loonen et al., 2019).

The usage of drugs which inactivate 5-HT receptors may decrease the difference between different genotypes corresponding to more or less activity of the corresponding receptor product. As several SGAs have significant affinity to HTR2A and somewhat less often to HTR2C, the usage of SGAs may obscure possible associations. This was apparently not true for *HTR2A* variants in our study. Excluding patients who were using HTR2A antagonists hardly changed the observed associations with TD (data not shown). This was also true for rs1928040, although significance was lost, apparently due to decreasing statistical power. This finding might also result in a certain level of doubt about the functional consequences of this rs1928040 variant. Excluding patients who were using HTR2C antagonizing SGAs, however, changed the magnitude of the associations. Due to the low number of the remaining patients, no statistically significant differences were found, but the size of the association between the G allele and the presence of total and orofacial TD increased to a major extent in the 102 studied (hemizygotic) men and decreased in the 144 remaining women after excluding HTR2C antagonist users. Our findings in men correspond to the prediction in Loonen et al. (2019).

The most important limitation of our study is that our finding cannot be applied to the identification of rs1928040 and rs1801412 as biomarkers because we assessed a possible association with a total of 24 variants of 7 *HTR* genes. We wanted to use our genotyping data to confirm or falsify our predefined hypothesis about the possible role of HTR2 in mediating “classical” TD symptoms. We did not find evidence supporting a possible role for HTR2A, but the effect of excluding patients who were using HTR2C antagonists indicates that HTR2C may have a specific role in developing (especially orofacial) TD. The number of male and female patients, who remain after excluding persons using HTR2C antagonists, is small which limits the reliability of this conclusion. Our findings should be verified in a prospective study of (hemizygous) male patients who are only using classical antipsychotics (or benzamides) devoid of HTR2 affinity. A third limitation would be that the treatment history of our patients cannot be adequately assessed. It should be emphasized that our findings are at least partly related to acute effects on the severity of the symptoms of TD (reflected by its prevalence). HTR2C could also have a role in the pathogenesis (related to its incidence) of this movement disorder, but this cannot be estimated.

## Conclusion

In this study we obtained evidence for an association between *HTR2A* polymorphism rs1928040 and *HTR2C* variant rs1801412 and particularly the orofacial form of tardive dyskinesia. Excluding patients who were using 5-HT2 antagonists suggests that HTR2C variety has functional consequences which may indicate a role of this receptor in modulating the severity of TD. However, this hypothesis needs verification in a larger group of male patients who are not using HTR2 antagonists.

## Data Availability Statement

Data are available from Prof. Dr. Svetlana A. Ivanova (ivanovaniipz@gmail.com) on reasonable request and with permission of MHRI.

## Ethics Statement

The studies involving human participants (protocol N63/7.2014) were reviewed and approved by the Local Bioethics Committee of the Mental Health Research Institute. The patients/participants provided their written informed consent to participate in this study.

## Author Contributions

SI and AL designed and supervised the study. EK collected the clinical information. IP and DP isolated DNA and genotyped the samples. AS and NB supervised the clinical work. SI, AL, and BW supervised the technical work. IP, SI, and AL designed and carried out the statistical analysis. AL wrote the first draft of manuscript. IP, OF, SI, and BW commented on this draft and contributed to the final manuscript.

## Conflict of Interest

The authors declare that the research was conducted in the absence of any commercial or financial relationships that could be construed as a potential conflict of interest.
