# Supplementary material for: 5-Hydroxytryptamine Receptors and Tardive Dyskinesia in Schizophrenia
Source: Front Mol Neurosci. 2020 Apr 24;13:63. doi: 10.3389/fnmol.2020.00063 (PMC7193905; doi:10.3389/fnmol.2020.00063)
Supplement: Supplementary file 1 [file Data_Sheet_1.PDF]

Supplementary table 1

Details of the selected SNPs

Gene	SNP	Chromosome	Chromosome position	Alleles	Minor allele frequency, %	HWE
HTR1A	rs6295	5	63962738	G/C	47.3	0.447
HTR1A	rs1364043	5	63955024	T/G	24.7	0.525
HTR1A	rs10042486	5	63965502	C/T	47.5	0.394
HTR1A	rs1800042	5	63960902	G/A	0.3	1.000**
HTR1A	rs749099	5	63958009	G/A	47.3	0.394
HTR1B	rs6298	6	77463275	C/T	26.6	0.717
HTR1B	rs6296	6	77462543	G/C	26.8	0.904
HTR1B	rs130058	6	77463564	A/T	25.6	0.898
HTR2A	rs6311	13	46897343	C/T	33.9	0.833
HTR2A	rs6313	13	46895805	C/T	33.7	0.833
HTR2A	rs6314	13	46834899	C/T	4.9	0.613**
HTR2A	rs7997012	13	46837850	A/G	49.4	0.156
HTR2A	rs1928040	13	46873101	C/T	41.9	0.103
HTR2A	rs9316233	13	46859220	C/G	21.9	0.315
HTR2A	rs2224721	13	46858019	C/A	25	0.165
HTR2A	rs6312	13	46896689	A/G	5	0.615
HTR2C	rs6318	X	114731326	G/C	10.9	0.000*
HTR2C	rs5946189	X	114837657	T/C	10.5	0.000*
HTR2C	rs569959	X	114585887	A/G	28.5	0.000*
HTR2C	rs17326429	X	114591899	G/A	15.5	0.000*
HTR2C	rs4911871	X	114762580	A/G	21.9	0.000*
HTR2C	rs3813929	X	114584047	C/T	15.2	0.000*
HTR2C	rs1801412	X	114908141	T/G	5	0.000*
HTR2C	rs12858300	X	114662932	G/C	4.4	0.000**
HTR3A	rs1062613	11	113975284	C/T	21.9	0.774
HTR3A	rs33940208	11	113975355	C/T	2	1.000**
HTR3A	rs1176713	11	113989703	T/C	23.3	0.353
HTR3B	rs1176744	11	113932306	G/T	12.8	0.005**
HTR6	rs1805054	1	19666020	C/T	21.3	0.887

Notes:

* - excluded from primary Hardy-Weinberg equilibrium testing because located in X-chromosome and separation by sex is needed;

** - excluded from analysis permanently due to minor allele frequency less than 5% or Hardy-Weinberg equilibrium test not passed ($p<0.05$).

Supplementary table 2

Results of association analysis for genotypes and alleles between groups of patients with total diagnosis of tardive dyskinesia (based on AIMS items 1-7) and without tardive dyskinesia.

SNP	Genotypes / alleles	Patients with total TD, %	Patients without TD, %	OR		χ^2	P
				Value	95% CI		
rs6295	GG	31 (25.8%)	97 (29.8%)	0.82	0.51 – 1.32	1.360	0.507
	GC	63 (52.5%)	151 (46.3%)	1.28	0.84 – 1.95		
	CC	26 (21.7%)	78 (23.9%)	0.88	0.53 – 1.45		
	G	0.521	0.529	0.97	0.72 – 1.30	0.05	0.83
	C	0.479	0.471	1.03	0.77 – 1.39		
rs1364043	GG	10 (8.3%)	20 (6.1%)	1.40	0.63 – 3.07	3.564	0.168
	GT	35 (29.2%)	126 (38.5%)	0.66	0.42 – 1.03		
	TT	75 (62.5%)	181 (55.4%)	1.34	0.88 – 2.06		
	G	0.229	0.254	0.87	0.62 – 1.24	0.57	0.45
	T	0.771	0.746	1.14	0.81 – 1.62		
rs10042486	CC	30 (24.8%)	98 (30.0%)	0.77	0.48 – 1.24	1.867	0.393
	CT	64 (52.9%)	150 (45.9%)	1.32	0.87 – 2.01		
	TT	27 (22.3%)	79 (24.2%)	0.90	0.55 – 1.48		
	C	0.512	0.529	0.94	0.70 – 1.26	0.20	0.66
	T	0.488	0.471	1.07	0.80 – 1.44		
rs749099	AA	27 (22.3%)	78 (23.9%)	0.92	0.56 – 1.51	1.311	0.519
	AG	63 (52.1%)	151 (46.2%)	1.27	0.83 – 1.92		
	GG	31 (25.6%)	98 (30.0%)	0.80	0.50 – 1.29		
	A	0.483	0.469	1.06	0.79 – 1.42	0.14	0.71
	G	0.517	0.531	0.95	0.70 – 1.27		
rs6298	CC	68 (56.2%)	175 (53.5%)	1.11	0.73 – 1.70	1.372	0.504
	CT	42 (34.7%)	130 (39.8%)	0.81	0.52 – 1.24		
	TT	11 (9.1%)	22 (6.7%)	1.39	0.65 – 2.95		
	C	0.736	0.734	1.01	0.72 – 1.41	0.01	0.96
	T	0.264	0.266	0.99	0.71 – 1.39		
rs6296	CC	11 (9.2%)	22 (6.7%)	1.40	0.66 – 2.98	1.497	0.473
	CG	42 (35.0%)	132 (40.4%)	0.80	0.51 – 1.23		
	GG	67 (55.8%)	173 (52.9%)	1.13	0.74 – 1.71		
	C	0.267	0.269	0.99	0.71 – 1.38	0.01	0.94
	G	0.733	0.731	1.01	0.72 – 1.41		
rs130058	TT	11 (9.7%)	17 (5.6%)	1.81	0.82 – 4.00	2.391	0.303
	TA	43 (38.1%)	114 (37.6%)	1.02	0.65 – 1.59		
	AA	59 (52.2%)	172 (56.8%)	0.83	0.54 – 1.28		
	T	0.288	0.244	1.25	0.89 – 1.76	1.63	0.2
	A	0.712	0.756	0.80	0.57 – 1.13		
rs6311	TT	16 (13.3%)	36 (11.0%)	1.24	0.66 – 2.33	0.529	0.768
	TC	51 (42.5%)	147 (45.1%)	0.90	0.59 – 1.37		
	CC	53 (44.2%)	143 (43.9%)	1.01	0.66 – 1.54		
	T	0.346	0.336	1.05	0.77 – 1.43	0.08	0.78
	C	0.654	0.664	0.96	0.70 – 1.31		
rs6313	CC	54 (44.6%)	144 (44.0%)	1.02	0.67 – 1.56	0.537	0.764
	CT	51 (42.1%)	147 (45.0%)	0.89	0.59 – 1.36		
	TT	16 (13.2%)	36 (11.0%)	1.23	0.66 – 2.31		
	C	0.657	0.665	0.96	0.71 – 1.32	0.05	0.82
	T	0.343	0.335	1.04	0.76 – 1.42		
rs7997012	AA	30 (25.0%)	76 (23.4%)	1.09	0.67 – 1.78	0.362	0.835
	AG	65 (54.2%)	173 (53.2%)	1.04	0.68 – 1.58		
	GG	25 (20.8%)	76 (23.4%)	0.86	0.52 – 1.44		

	A	0.521	0.500	1.09	0.81 – 1.46	0.30	0.58
	G	0.479	0.500	0.92	0.68 – 1.24		
rs1928040	See Table 2						
rs9316233	GG	5 (4.5%)	11 (3.6%)	1.24	0.42 – 3.65	0.186	0.911
	GC	41 (36.6%)	109 (36.0%)	1.03	0.66 – 1.61		
	CC	66 (58.9%)	183 (60.4%)	0.94	0.61 – 1.46		
	G	0.228	0.216	1.07	0.74 – 1.54	0.13	0.72
	C	0.772	0.784	0.94	0.65 – 1.35		
rs2224721	CC	65 (54.2%)	180 (55.2%)	0.96	0.63 – 1.46	0.309	0.857
	CA	50 (41.7%)	129 (39.6%)	1.09	0.71 – 1.67		
	AA	5 (4.2%)	17 (5.2%)	0.79	0.29 – 2.19		
	C	0.750	0.750	1.00	0.71 – 1.41	0.01	1
	A	0.250	0.250	1.00	0.71 – 1.41		
rs6312	GG	0 (0.0%)	0 (0.0%)	2.72	0.05 – 138.05	0.006	0.941
	GA	12 (10.1%)	32 (9.8%)	1.03	0.51 – 2.07		
	AA	107 (89.9%)	293 (90.2%)	0.97	0.48 – 1.96		
	G	0.050	0.049	1.03	0.52 – 2.03	0.01	0.94
	A	0.950	0.951	0.98	0.49 – 1.93		
rs1062613	CC	65 (57.5%)	190 (62.7%)	0.81	0.52 – 1.25	0.951	0.622
	CT	42 (37.2%)	98 (32.3%)	1.24	0.79 – 1.94		
	TT	6 (5.3%)	15 (5.0%)	1.08	0.41 – 2.85		
	C	0.761	0.789	0.85	0.59 – 1.23	0.74	0.39
	T	0.239	0.211	1.17	0.82 – 1.68		
rs1176713	CC	6 (5.0%)	22 (6.7%)	0.73	0.29 – 1.85	0.453	0.797
	CT	41 (34.2%)	111 (33.9%)	1.01	0.65 – 1.57		
	TT	73 (60.8%)	194 (59.3%)	1.06	0.69 – 1.63		
	C	0.221	0.237	0.91	0.64 – 1.30	0.26	0.61
	T	0.779	0.763	1.10	0.77 – 1.56		
rs1805054	TT	5 (4.2%)	14 (4.3%)	0.97	0.34 – 2.75	0.013	0.993
	TC	40 (33.6%)	110 (34.1%)	0.98	0.63 – 1.53		
	CC	74 (62.2%)	199 (61.6%)	1.02	0.66 – 1.58		
	T	0.210	0.214	0.98	0.68 – 1.41	0.01	0.91
	C	0.790	0.786	1.02	0.71 – 1.47		

Note: significancy ($p < 0.05$) indicated in bold

Supplementary table 3

Results of association analysis for genotypes and alleles between groups of patients with diagnosis of orofacial tardive dyskinesia (based on AIMS items 1-4) and without tardive dyskinesia.

SNP	Genotypes / alleles	Patients with orofacial TD, %	Patients without TD, %	OR		χ^2	P
				Value	95% CI		
rs6295	GG	30 (27.5%)	98 (29.1%)	0.93	0.57 – 1.50	1.907	0.385
	GC	58 (53.2%)	156 (46.3%)	1.32	0.86 – 2.03		
	CC	21 (19.3%)	83 (24.6%)	0.73	0.43 – 1.25		
	G	0.541	0.522	1.08	0.79 – 1.47	0.24	0.62
	C	0.459	0.478	0.93	0.68 – 1.26		
rs1364043	GG	8 (7.4%)	22 (6.5%)	1.15	0.50 – 2.67	2.522	0.283
	GT	32 (29.6%)	129 (38.1%)	0.69	0.43 – 1.09		
	TT	68 (63.0%)	188 (55.5%)	1.37	0.87 – 2.13		
	G	0.222	0.255	0.83	0.58 – 1.20	0.96	0.33
	T	0.778	0.745	1.20	0.83 – 1.73		
rs10042486	CC	29 (26.6%)	99 (29.2%)	0.99	0.61 – 1.62	2.591	0.274
	CT	59 (54.1%)	155 (45.7%)	1.71	1.09 – 2.68		
	TT	12 (19.3%)	85 (25.1%)	0.41	0.21 – 0.78		
	C	0.585	0.521	1.30	0.94 – 1.79	2.57	0.11
	T	0.415	0.479	0.77	0.56 – 1.06		
rs749099	AA	21 (19.3%)	84 (24.8%)	0.72	0.42 – 1.24	2.044	0.360
	AG	58 (53.2%)	156 (46.0%)	1.33	0.87 – 2.06		
	GG	30 (27.5%)	99 (29.2%)	0.92	0.57 – 1.49		
	A	0.459	0.478	0.93	0.68 – 1.26	0.24	0.62
	G	0.541	0.522	1.08	0.80 – 1.47		
rs6298	CC	63 (57.8%)	180 (53.1%)	1.21	0.78 – 1.87	1.233	0.540
	CT	37 (33.9%)	135 (39.8%)	0.78	0.49 – 1.22		
	TT	9 (8.3%)	24 (7.1%)	1.18	0.53 – 2.62		
	C	0.748	0.730	1.10	0.77 – 1.55	0.26	0.61
	T	0.252	0.270	0.91	0.64 – 1.29		
rs6296	CC	9 (8.3%)	24 (7.1%)	1.19	0.54 – 2.65	1.337	0.512
	CG	37 (34.3%)	137 (40.4%)	0.77	0.49 – 1.21		
	GG	62 (57.4%)	178 (52.5%)	1.22	0.79 – 1.89		
	C	0.255	0.273	0.91	0.64 – 1.29	0.28	0.6
	G	0.745	0.727	1.10	0.77 – 1.56		
rs130058	TT	10 (9.7%)	18 (5.8%)	1.76	0.79 – 3.95	3.030	0.220
	TA	42 (40.8%)	115 (36.7%)	1.19	0.75 – 1.87		
	AA	51 (49.5%)	180 (57.5%)	0.72	0.46 – 1.13		
	T	0.301	0.241	1.35	0.95 – 1.92	2.91	0.09
	A	0.699	0.759	0.74	0.52 – 1.05		
rs6311	TT	15 (13.9%)	37 (10.9%)	1.31	0.69 – 2.50	1.460	0.482
	TC	43 (39.8%)	155 (45.9%)	0.78	0.50 – 1.21		
	CC	50 (46.3%)	146 (43.2%)	1.13	0.73 – 1.75		
	T	0.338	0.339	1.00	0.72 – 1.38	0.01	0.98
	C	0.662	0.661	1.00	0.73 – 1.39		
rs6313	CC	51 (46.8%)	147 (43.4%)	1.15	0.74 – 1.77	1.529	0.465
	CT	43 (39.4%)	155 (45.7%)	0.77	0.50 – 1.20		
	TT	15 (13.8%)	37 (10.9%)	1.30	0.68 – 2.48		
	C	0.665	0.662	1.01	0.73 – 1.40	0.01	0.94
	T	0.335	0.338	0.99	0.71 – 1.36		
rs7997012	AA	27 (25.0%)	79 (23.4%)	1.09	0.66 – 1.80	0.208	0.901
	AG	58 (53.7%)	180 (53.4%)	1.01	0.66 – 1.56		
	GG	23 (21.3%)	78 (23.1%)	0.90	0.53 – 1.52		
	A	0.519	0.501	1.07	0.79 – 1.45	0.19	0.66
	G	0.481	0.499	0.93	0.69 – 1.27		

rs1928040	See Table 2						
rs9316233	GG	5 (5.0%)	11 (3.5%)	1.43	0.49 – 4.23	1.319	0.517
	GC	40 (39.6%)	110 (35.0%)	1.22	0.77 – 1.93		
	CC	56 (55.4%)	193 (61.5%)	0.78	0.50 – 1.23		
	G	0.248	0.210	1.24	0.85 – 1.79	1.24	0.26
	C	0.752	0.790	0.81	0.56 – 1.17		
rs2224721	CC	57 (52.3%)	188 (55.8%)	0.87	0.56 – 1.34	0.538	0.764
	CA	47 (43.1%)	132 (39.2%)	1.18	0.76 – 1.82		
	AA	5 (4.6%)	17 (5.0%)	0.90	0.33 – 2.51		
	C	0.739	0.754	0.92	0.65 – 1.31	0.20	0.65
	A	0.261	0.246	1.08	0.76 – 1.54		
rs6312	GG	0 (0.0%)	0 (0.0%)	3.14	0.06 – 159.19	0.269	0.604
	GA	12 (11.2%)	32 (9.5%)	1.20	0.60 – 2.43		
	AA	95 (88.8%)	305 (90.5%)	0.83	0.41 – 1.68		
	G	0.056	0.047	1.19	0.60 – 2.36	0.25	0.61
	A	0.944	0.953	0.84	0.42 – 1.66		
rs1062613	CC	59 (57.8%)	196 (62.4%)	0.83	0.52 – 1.30	0.790	0.674
	CT	38 (37.3%)	102 (32.5%)	1.23	0.77 – 1.97		
	TT	5 (4.9%)	16 (5.1%)	0.96	0.34 – 2.69		
	C	0.765	0.787	0.88	0.61 – 1.28	0.43	0.51
	T	0.235	0.213	1.13	0.78 – 1.65		
rs1176713	CC	5 (4.6%)	23 (6.8%)	0.67	0.25 – 1.80	0.655	0.721
	CT	37 (34.3%)	115 (33.9%)	1.02	0.64 – 1.60		
	TT	66 (61.1%)	201 (59.3%)	1.08	0.69 – 1.68		
	C	0.218	0.237	0.89	0.62 – 1.29	0.36	0.55
	T	0.782	0.763	1.12	0.77 – 1.62		
rs1805054	TT	4 (3.7%)	15 (4.5%)	0.83	0.27 – 2.55	0.230	0.891
	TC	38 (35.5%)	112 (33.4%)	1.10	0.69 – 1.73		
	CC	65 (60.7%)	208 (62.1%)	0.94	0.60 – 1.48		
	T	0.215	0.212	1.02	0.70 – 1.48	0.01	0.93
	C	0.785	0.788	0.98	0.67 – 1.43		

Note: significancy ($p < 0.05$) indicated in bold

Supplementary table 4

Results of association analysis for genotypes and alleles between groups of patients with a diagnosis of limb-truncal tardive dyskinesia (based on AIMS items 5-7) and without tardive dyskinesia.

SNP	Genotypes / alleles	Patients with limbtruncal TD, %	Patients without TD, %	OR		χ^2	P
				Value	95% CI		
rs6295	GG	19 (29.2%)	109 (28.6%)	1.03	0.58 – 1.84	0.490	0.783
	GC	33 (50.8%)	181 (47.5%)	1.14	0.67 – 1.93		
	CC	13 (20.0%)	91 (23.9%)	0.80	0.42 – 1.53		
	G	0.546	0.524	1.09	0.75 – 1.59	0.23	0.63
	C	0.454	0.476	0.91	0.63 – 1.33		
rs1364043	GG	5 (7.8%)	25 (6.5%)	1.21	0.45 – 3.30	2.899	0.235
	GT	17 (26.6%)	144 (37.6%)	0.60	0.33 – 1.09		
	TT	42 (65.6%)	214 (55.9%)	1.51	0.87 – 2.62		
	G	0.211	0.253	0.79	0.50 – 1.24	1.06	0.3
	T	0.789	0.747	1.27	0.81 – 2.00		
rs10042486	CC	18 (27.7%)	110 (28.7%)	0.95	0.53 – 1.71	0.779	0.677
	CT	34 (52.3%)	180 (47.0%)	1.24	0.73 – 2.09		
	TT	13 (20.0%)	93 (24.3%)	0.78	0.41 – 1.49		
	C	0.538	0.522	1.07	0.74 – 1.55	0.12	0.73
	T	0.462	0.478	0.94	0.65 – 1.36		
rs749099	AA	13 (20.0%)	92 (24.0%)	0.79	0.41 – 1.52	0.532	0.767
	AG	33 (50.8%)	181 (47.3%)	1.15	0.68 – 1.95		
	GG	19 (29.2%)	110 (28.7%)	1.03	0.57 – 1.83		
	A	0.454	0.477	0.91	0.63 – 1.33	0.23	0.63
	G	0.546	0.523	1.10	0.75 – 1.59		
rs6298	CC	37 (56.9%)	206 (53.8%)	1.14	0.67 – 1.93	0.882	0.643
	CT	25 (38.5%)	147 (38.4%)	1.00	0.58 – 1.72		
	TT	3 (4.6%)	30 (7.8%)	0.57	0.17 – 1.92		
	C	0.762	0.730	1.18	0.77 – 1.82	0.58	0.45
	T	0.238	0.270	0.85	0.55 – 1.30		
rs6296	CC	3 (4.7%)	30 (7.8%)	0.58	0.17 – 1.96	0.826	0.662
	CG	25 (39.1%)	149 (38.9%)	1.01	0.59 – 1.73		
	GG	36 (56.3%)	204 (53.3%)	1.13	0.66 – 1.92		
	C	0.242	0.273	0.85	0.55 – 1.32	0.52	0.47
	G	0.758	0.727	1.17	0.76 – 1.81		
rs130058	TT	7 (11.7%)	21 (5.9%)	2.11	0.85 – 5.20	2.902	0.234
	TA	20 (33.3%)	137 (38.5%)	0.80	0.45 – 1.42		
	AA	33 (55.0%)	198 (55.6%)	0.98	0.56 – 1.69		
	T	0.283	0.251	1.18	0.76 – 1.81	0.55	0.46
	A	0.717	0.749	0.85	0.55 – 1.31		
rs6311	TT	10 (15.6%)	42 (11.0%)	1.50	0.71 – 3.16	1.248	0.536
	TC	26 (40.6%)	172 (45.0%)	0.84	0.49 – 1.43		
	CC	28 (43.8%)	168 (44.0%)	0.99	0.58 – 1.69		
	T	0.359	0.335	1.11	0.75 – 1.65	0.29	0.59
	C	0.641	0.665	0.90	0.61 – 1.33		
rs6313	CC	29 (44.6%)	169 (44.1%)	1.02	0.60 – 1.73	1.241	0.538
	CT	26 (40.0%)	172 (44.9%)	0.82	0.48 – 1.40		
	TT	10 (15.4%)	42 (11.0%)	1.48	0.70 – 3.11		
	C	0.646	0.666	0.92	0.62 – 1.35	0.19	0.66
	T	0.354	0.334	1.09	0.74 – 1.61		
rs7997012	AA	18 (27.7%)	88 (23.2%)	1.27	0.70 – 2.30	1.083	0.582
	AG	35 (53.8%)	203 (53.4%)	1.02	0.60 – 1.72		
	GG	12 (18.5%)	89 (23.4%)	0.74	0.38 – 1.45		
	A	0.546	0.499	1.21	0.83 – 1.76	1.00	0.32
	G	0.454	0.501	0.83	0.57 – 1.20		

rs1928040	See Table 2						
rs9316233	GG	2 (3.4%)	14 (3.9%)	0.86	0.19 – 3.87	2.759	0.252
	GC	27 (45.8%)	123 (34.6%)	1.60	0.92 – 2.79		
	CC	30 (50.8%)	219 (61.5%)	0.65	0.37 – 1.13		
	G	0.263	0.212	1.32	0.85 – 2.07	1.52	0.22
	C	0.737	0.788	0.76	0.48 – 1.18		
rs2224721	CC	33 (50.8%)	212 (55.6%)	0.82	0.49 – 1.39	3.100	0.212
	CA	31 (47.7%)	148 (38.8%)	1.44	0.85 – 2.44		
	AA	1 (1.5%)	21 (5.5%)	0.27	0.04 – 2.03		
	C	0.746	0.751	0.98	0.64 – 1.50	0.01	0.91
	A	0.254	0.249	1.02	0.67 – 1.57		
rs6312	GG	0 (0.0%)	0 (0.0%)	5.79	0.11 – 294.58	0.063	0.802
	GA	7 (10.8%)	37 (9.8%)	1.12	0.47 – 2.62		
	AA	58 (89.2%)	342 (90.2%)	0.90	0.38 – 2.11		
	G	0.054	0.049	1.11	0.48 – 2.54	0.06	0.81
	A	0.946	0.951	0.90	0.39 – 2.07		
rs1062613	CC	34 (56.7%)	221 (62.1%)	0.80	0.46 – 1.39	1.493	0.474
	CT	24 (40.0%)	116 (32.6%)	1.38	0.79 – 2.42		
	TT	2 (3.3%)	19 (5.3%)	0.61	0.14 – 2.70		
	C	0.767	0.784	0.91	0.57 – 1.44	0.17	0.68
	T	0.233	0.216	1.10	0.70 – 1.75		
rs1176713	CC	4 (6.3%)	24 (6.3%)	1.00	0.33 – 2.98	0.644	0.725
	CT	19 (29.7%)	133 (34.7%)	0.79	0.45 – 1.41		
	TT	41 (64.1%)	226 (59.0%)	1.24	0.71 – 2.15		
	C	0.211	0.236	0.86	0.55 – 1.36	0.39	0.53
	T	0.789	0.764	1.16	0.73 – 1.83		
rs1805054	TT	1 (1.5%)	18 (4.8%)	0.31	0.04 – 2.38	1.648	0.439
	TC	21 (32.3%)	129 (34.2%)	0.92	0.52 – 1.61		
	CC	43 (66.2%)	230 (61.0%)	1.25	0.72 – 2.17		
	T	0.177	0.219	0.77	0.47 – 1.24	1.16	0.28
	C	0.823	0.781	1.30	0.80 – 2.11		

Note: significancy ($p < 0.05$) indicated in bold

Supplementary Table 5

Results of association analysis for *5-HT2c receptor* gene genotypes and alleles between groups of patients with total tardive dyskinesia (based on AIMS items 1-7) and without tardive dyskinesia divided by sex.

SNP	Genotypes / alleles	Patients with total TD, %	Patients without TD, %	OR		χ^2	P
				Value	95% CI		
rs6318 male	G	62 (87.3%)	126 (87.5%)	0.98	0.54 – 1.80	0.001	0.96
	C	9 (12.7%)	18 (11.8%)	1.02	0.55 – 1.86		
rs6318 female	GG	38 (79.2%)	143 (81.7%)	0.85	0.38 – 1.88	0.504	0.777
	GC	10 (20.8%)	31 (17.7%)	1.22	0.55 – 2.71		
	CC	0 (0.0%)	1 (0.6%)	1.20	0.05 – 29.91		
	G	0.896	0.906	0.90	0.42 – 1.89	0.08	0.77
	C	0.104	0.094	1.12	0.53 – 2.36		
rs5946189 male	T	62 (87.3%)	125 (88.0%)	0.94	0.51 – 1.73	0.04	0.83
	C	9 (12.7%)	17 (12.0%)	1.07	0.58 – 1.97		
rs5946189 female	TT	40 (80.0%)	144 (82.3%)	0.86	0.39 – 1.91	0.489	0.783
	TC	10 (20.0%)	30 (17.1%)	1.21	0.54 – 2.68		
	CC	0 (0.0%)	1 (0.6%)	1.15	0.05 – 28.71		
	T	0.900	0.909	0.91	0.43 – 1.91	0.07	0.8
	C	0.100	0.091	1.10	0.52 – 2.33		
rs569959 male	G	18 (25.4%)	44 (30.8%)	0.76	0.49 – 1.20	1.35	0.24
	A	53 (74.6%)	99 (69.2%)	1.31	0.83 – 2.06		
rs569959 female	GG	3 (6.0%)	8 (4.5%)	1.34	0.34 – 5.25	0.186	0.911
	GA	23 (46.0%)	81 (46.0%)	1.00	0.53 – 1.88		
	AA	24 (48.0%)	87 (49.4%)	0.94	0.50 – 1.77		
	G	0.290	0.276	1.07	0.66 – 1.75	0.08	0.78
	A	0.710	0.724	0.93	0.57 – 1.52		
rs17326429 male	A	9 (12.7%)	20 (14.1%)	0.89	0.49 – 1.61	0.16	0.69
	G	62 (87.3%)	122 (85.9%)	1.13	0.62 – 2.05		
rs17326429 female	AA	1 (2.0%)	3 (1.7%)	1.20	0.12 – 11.81	0.145	0.930
	AG	14 (28.6%)	55 (31.3%)	0.88	0.44 – 1.77		
	GG	34 (69.4%)	118 (67.0%)	1.11	0.56 – 2.21		
	A	0.163	0.173	0.93	0.51 – 1.70	0.05	0.82
	G	0.837	0.827	1.07	0.59 – 1.96		
rs4911871 male	G	16 (22.5%)	32 (22.2%)	1.02	0.63 – 1.65	0.01	0.94
	A	55 (77.5%)	112 (77.8%)	0.98	0.61 – 1.59		
rs4911871 female	GG	2 (4.1%)	4 (2.3%)	1.82	0.32 – 10.24	1.213	0.545
	GA	20 (40.8%)	61 (34.9%)	1.29	0.67 – 2.47		
	AA	27 (55.1%)	110 (62.9%)	0.73	0.38 – 1.38		
	G	0.245	0.197	1.32	0.78 – 2.24	1.06	0.3
	A	0.755	0.803	0.76	0.45 – 1.29		
rs3813929 male	C	62 (88.6%)	124 (86.7%)	1.19	0.64 – 2.21	0.29	0.59
	T	8 (11.4%)	19 (13.3%)	0.84	0.45 – 1.57		
rs3813929 female	CC	35 (70.0%)	118 (67.0%)	1.15	0.58 – 2.27	0.204	0.903
	CT	14 (28.0%)	55 (31.3%)	0.86	0.43 – 1.71		
	TT	1 (2.0%)	3 (1.7%)	1.18	0.12 – 11.57		
	C	0.840	0.827	1.10	0.60 – 2.01	0.10	0.76
	T	0.160	0.173	0.91	0.50 – 1.66		
rs1801412 male	G	4 (5.7%)	7 (4.9%)	1.17	0.48 – 2.86	0.12	0.73
	T	66 (94.3%)	135 (95.1%)	0.86	0.35 – 2.09		
rs1801412 female	See Table 3						

Note: significancy ($p < 0.05$) indicated in bold

Supplementary Table 6

Results of association analysis for *5-HT2c receptor* gene genotypes and alleles between groups of patients with orofacial type of tardive dyskinesia (based on AIMS items 1-4) and without tardive dyskinesia divided by sex

SNP	Genotypes / alleles	Patients with orofacial TD, %	Patients without TD, %	OR		χ^2	P
				Value	95% CI		
rs6318 male	G	57 (86.4%)	131 (87.9%)	0.87	0.47 – 1.60	0.20	0.65
	C	9 (13.6%)	18 (12.1%)	1.15	0.63 – 2.11		
rs6318 female	GG	33 (80.5%)	148 (81.3%)	0.95	0.40 – 2.23	0.263	0.877
	GC	8 (19.5%)	33 (18.1%)	1.09	0.46 – 2.59		
	CC	0 (0.0%)	1 (0.5%)	1.46	0.06 – 36.43		
	G	0.902	0.904	0.98	0.44 – 2.21	0.001	0.97
	C	0.098	0.096	1.02	0.45 – 2.28		
rs5946189 male	T	57 (86.4%)	130 (88.4%)	0.83	0.45 – 1.53	0.36	0.55
	C	9 (13.6%)	17 (11.6%)	1.21	0.65 – 2.23		
rs5946189 female	TT	35 (81.4%)	149 (81.9%)	0.97	0.41 – 2.28	0.258	0.879
	TC	8 (18.6%)	32 (17.6%)	1.07	0.45 – 2.53		
	CC	0 (0.0%)	1 (0.5%)	1.39	0.06 – 34.73		
	T	0.907	0.907	1.00	0.45 – 2.26	0.001	0.99
	C	0.093	0.093	1.00	0.44 – 2.23		
rs569959 male	G	17 (25.8%)	45 (30.4%)	0.79	0.50 – 1.26	0.96	0.33
	A	49 (74.2%)	103 (69.6%)	1.26	0.79 – 2.00		
rs569959 female	GG	2 (4.7%)	9 (4.9%)	0.94	0.20 – 4.53	0.009	0.996
	GA	20 (46.5%)	84 (45.9%)	1.02	0.53 – 1.99		
	AA	21 (48.8%)	90 (49.2%)	0.99	0.51 – 1.92		
	G	0.279	0.279	1.00	0.59 – 1.69	0.001	0.99
	A	0.721	0.721	1.00	0.59 – 1.69		
rs17326429 male	A	8 (12.1%)	21 (14.3%)	0.83	0.45 – 1.53	0.36	0.55
	G	58 (87.9%)	126 (85.7%)	1.21	0.65 – 2.24		
rs17326429 female	AA	0 (0.0%)	4 (2.2%)	0.47	0.02 – 8.88	0.936	0.626
	AG	13 (31.0%)	56 (30.6%)	1.02	0.49 – 2.10		
	GG	29 (69.0%)	123 (67.2%)	1.09	0.53 – 2.24		
	A	0.155	0.175	0.86	0.45 – 1.65	0.19	0.66
	G	0.845	0.825	1.16	0.60 – 2.22		
rs4911871 male	G	14 (21.2%)	34 (22.8%)	0.91	0.55 – 1.50	0.14	0.71
	A	52 (78.8%)	115 (77.2%)	1.10	0.67 – 1.81		
rs4911871 female	GG	1 (2.4%)	5 (2.7%)	0.86	0.10 – 7.59	1.847	0.397
	GA	19 (45.2%)	62 (34.1%)	1.60	0.81 – 3.16		
	AA	22 (52.4%)	115 (63.2%)	0.64	0.33 – 1.26		
	G	0.250	0.198	1.35	0.77 – 2.36	1.13	0.29
	A	0.750	0.802	0.74	0.42 – 1.29		
rs3813929 male	C	58 (87.9%)	128 (87.1%)	1.08	0.58 – 2.01	0.05	0.82
	T	8 (12.1%)	19 (12.9%)	0.93	0.50 – 1.73		
rs3813929 female	CC	30 (69.8%)	123 (67.2%)	1.13	0.55 – 2.31	0.975	0.614
	CT	13 (30.2%)	56 (30.6%)	0.98	0.48 – 2.02		
	TT	0 (0.0%)	4 (2.2%)	0.46	0.02 – 8.68		
	C	0.849	0.825	1.19	0.62 – 2.28	0.28	0.6
	T	0.151	0.175	0.84	0.44 – 1.61		
rs1801412 male	G	4 (6.2%)	7 (4.8%)	1.31	0.54 – 3.21	0.36	0.55
	T	61 (93.8%)	140 (95.2%)	0.76	0.31 – 1.86		
rs1801412 female	See Table 3						

Note: significancy ($p < 0.05$) indicated in bold

Supplementary Table 7

Results of association analysis for *5-HT2c receptor* gene genotypes and alleles between groups of patients with limb-truncal type of tardive dyskinesia (AIMS item 5-7) and without tardive dyskinesia divided by sex

SNP	Genotypes / alleles	Patients with limbtruncal TD, %	Patients without TD, %	OR		χ^2	p
				Value	95% CI		
rs6318 male	G	33 (89.2%)	155 (87.1%)	1.22	0.55 – 2.71	0.25	0.62
	C	4 (10.8%)	23 (12.9%)	0.82	0.37 – 1.81		
rs6318 female	GG	19 (73.1%)	162 (82.2%)	0.59	0.23 – 1.50	1.536	0.464
	GC	7 (26.9%)	34 (17.3%)	1.77	0.69 – 4.53		
	CC	0 (0.0%)	1 (0.5%)	2.47	0.10 – 62.25		
	G	0.865	0.909	0.65	0.27 – 1.54	0.99	0.32
	C	0.135	0.091	1.55	0.65 – 3.68		
rs5946189 male	T	33 (89.2%)	154 (87.5%)	1.18	0.53 – 2.62	0.16	0.69
	C	4 (10.8%)	22 (12.5%)	0.85	0.38 – 1.89		
rs5946189 female	TT	21 (75.0%)	163 (82.7%)	0.63	0.25 – 1.59	1.260	0.533
	TC	7 (25.0%)	33 (16.8%)	1.66	0.65 – 4.21		
	CC	0 (0.0%)	1 (0.5%)	2.30	0.09 – 57.79		
	T	0.875	0.911	0.68	0.29 – 1.62	0.76	0.38
	C	0.125	0.089	1.47	0.62 – 3.48		
rs569959 male	G	9 (24.3%)	53 (29.9%)	0.75	0.42 – 1.34	0.94	0.33
	A	28 (75.7%)	124 (70.1%)	1.33	0.75 – 2.37		
rs569959 female	GG	2 (7.1%)	9 (4.5%)	1.62	0.33 – 7.89	0.784	0.676
	GA	11 (39.3%)	93 (47.0%)	0.73	0.33 – 1.64		
	AA	15 (53.6%)	96 (48.5%)	1.23	0.55 – 2.71		
	G	0.268	0.280	0.94	0.50 – 1.77	0.04	0.85
	A	0.732	0.720	1.06	0.57 – 2.00		
rs17326429 male	A	5 (13.5%)	24 (13.6%)	0.99	0.48 – 2.06	0.001	0.98
	G	32 (86.5%)	152 (86.4%)	1.01	0.49 – 2.10		
rs17326429 female	AA	1 (3.6%)	3 (1.5%)	2.40	0.24 – 23.86	4.384	0.112
	AG	4 (14.3%)	65 (33.0%)	0.34	0.11 – 1.02		
	GG	23 (82.1%)	129 (65.5%)	2.42	0.88 – 6.66		
	A	0.107	0.180	0.55	0.23 – 1.32	1.85	0.17
	G	0.893	0.820	1.83	0.76 – 4.44		
rs4911871 male	G	7 (18.9%)	41 (23.0%)	0.78	0.41 – 1.47	0.60	0.44
	A	30 (81.1%)	137 (77.0%)	1.28	0.68 – 2.41		
rs4911871 female	GG	1 (3.7%)	5 (2.5%)	1.48	0.17 – 13.14	0.635	0.728
	GA	8 (29.6%)	73 (37.1%)	0.72	0.30 – 1.72		
	AA	18 (66.7%)	119 (60.4%)	1.31	0.56 – 3.07		
	G	0.185	0.211	0.85	0.41 – 1.76	0.19	0.67
	A	0.815	0.789	1.17	0.57 – 2.43		
rs3813929 male	C	32 (88.9%)	154 (87.0%)	1.19	0.54 – 2.65	0.19	0.66
	T	4 (11.1%)	23 (13.0%)	0.84	0.38 – 1.86		
rs3813929 female	CC	23 (82.1%)	130 (65.7%)	2.41	0.88 – 6.61	4.333	0.115
	CT	4 (14.3%)	65 (32.8%)	0.34	0.11 – 1.02		
	TT	1 (3.6%)	3 (1.5%)	2.41	0.24 – 23.98		
	C	0.893	0.821	1.82	0.75 – 4.41	1.81	0.18
	T	0.107	0.179	0.55	0.23 – 1.33		
rs1801412 male	G	2 (5.4%)	9 (5.1%)	1.05	0.35 – 3.21	0.01	0.93
	T	35 (94.6%)	166 (94.9%)	0.95	0.31 – 2.89		
rs1801412 female	See Table 3						

Supplementary Table 8

Results of association analysis for *5-HT2c receptor* gene genotypes and alleles between groups of all patients with tardive dyskinesia and without tardive dyskinesia divided by sex.

SNP / sex / TD type	Genotypes / alleles	Patients with TD, %	Patients without TD, %	OR value	OR 95% CI	χ^2	p
rs1801412 / male / total TD	G	4 (5.7%)	7 (4.9%)	1.17	0.48 – 2.86	0.12	0.73
	T	66 (94.3%)	135 (95.1%)	0.86	0.35 – 2.09		
rs1801412 / female / total TD	GG	0 (0.0%)	0 (0.0%)	3.57	0.07 – 182.00	4.882	**0.027**
	GT	7 (14.3%)	9 (5.1%)	3.09	1.09 – 8.79		
	TT	42 (85.7%)	167 (94.9%)	0.32	0.11 – 0.92		
	G	0.071	0.026	2.93	1.06 – 8.08	4.70	**0.03**
	T	0.929	0.974	0.34	0.12 – 0.94		
rs1801412 / male / orofacial TD	G	4 (6.2%)	7 (4.8%)	1.31	0.54 – 3.21	0.36	0.55
	T	61 (93.8%)	140 (95.2%)	0.76	0.31 – 1.86		
rs1801412 / female / orofacial TD	GG	0 (0.0%)	0 (0.0%)	4.32	0.08 – 220.72	7.138	**0.008**
	GT	7 (16.7%)	9 (4.9%)	3.87	1.35 – 11.08		
	TT	35 (83.3%)	174 (95.1%)	0.26	0.09 – 0.74		
	G	0.083	0.025	3.61	1.30 – 9.98	6.88	**0.009**
	T	0.917	0.975	0.28	0.10 – 0.77		
rs1801412 / male / limb-truncal TD	G	2 (5.4%)	9 (5.1%)	1.05	0.35 – 3.21	0.01	0.93
	T	35 (94.6%)	166 (94.9%)	0.95	0.31 – 2.89		
rs1801412 / female limb-truncal TD	GG	0 (0.0%)	0 (0.0%)	6.93	0.13 – 356.19	0.629	0.428
	GT	3 (10.7%)	13 (6.6%)	1.70	0.45 – 6.38		
	TT	25 (89.3%)	184 (93.4%)	0.59	0.16 – 2.21		
	G	0.054	0.033	1.66	0.46 – 6.01	0.61	0.44
	T	0.946	0.967	0.60	0.17 – 2.19		

Note: significancy ($p < 0.05$) indicated in bold

Supplementary Table 9

Results of association analysis for *5-HT2c receptor* gene genotypes and alleles between groups of patients with tardive dyskinesia and without tardive dyskinesia divided by sex excluding patients using a 5-HT2C receptor antagonist.

SNP / sex / TD type	Genotypes / alleles	Patients with TD, %	Patients without TD, %	OR		χ^2	p
				value	95% CI		
rs1801412 / male / total TD	G	3 (11.1%)	4 (5.3%)	2.22	0.73 – 6.72	2.07	0.15
	T	24 (88.9%)	71 (94.7%)	0.45	0.15 – 1.36		
rs1801412 / female / total TD	GG	0 (0.0%)	0 (0.0%)			0.320	0.572
	GT	2 (9.1%)	7 (5.9%)	1.60	0.31 – 8.26		
	TT	20 (90.9%)	112 (94.1%)	0.63	0.12 – 3.23		
	G	0.045	0.029	1.57	0.32 – 7.83	0.31	0.58
	T	0.955	0.971	0.64	0.13 – 3.17		
rs1801412 / male / orofacial TD	G	3 (12.5%)	4 (5.1%)	2.64	0.87 – 8.04	3.12	0.08
	T	21 (87.5%)	74 (94.9%)	0.38	0.12 – 1.15		
rs1801412 / female / orofacial TD	GG	0 (0.0%)	0 (0.0%)			0.937	0.333
	GT	2 (11.8%)	7 (5.6%)	2.23	0.42 – 11.73		
	TT	15 (88.2%)	117 (94.4%)	0.45	0.09 – 2.36		
	G	0.059	0.028	2.15	0.43 – 10.81	0.91	0.34
	T	0.941	0.972	0.46	0.09 – 2.33		
rs1801412 / male / limb-truncal TD	G	2 (13.3%)	5 (5.7%)	2.52	0.74 – 8.64	2.30	0.13
	T	13 (86.7%)	82 (94.3%)	0.40	0.12 – 1.36		
rs1801412 / female limb-truncal TD	GG	0 (0.0%)	0 (0.0%)			2.321	0.128
	GT	2 (16.7%)	7 (5.4%)	3.49	0.64 – 19.05		
	TT	10 (83.3%)	122 (94.6%)	0.29	0.05 – 1.57		
	G	0.083	0.027	3.26	0.64 – 16.65	2.24	0.13
	T	0.917	0.973	0.31	0.06 – 1.57		
